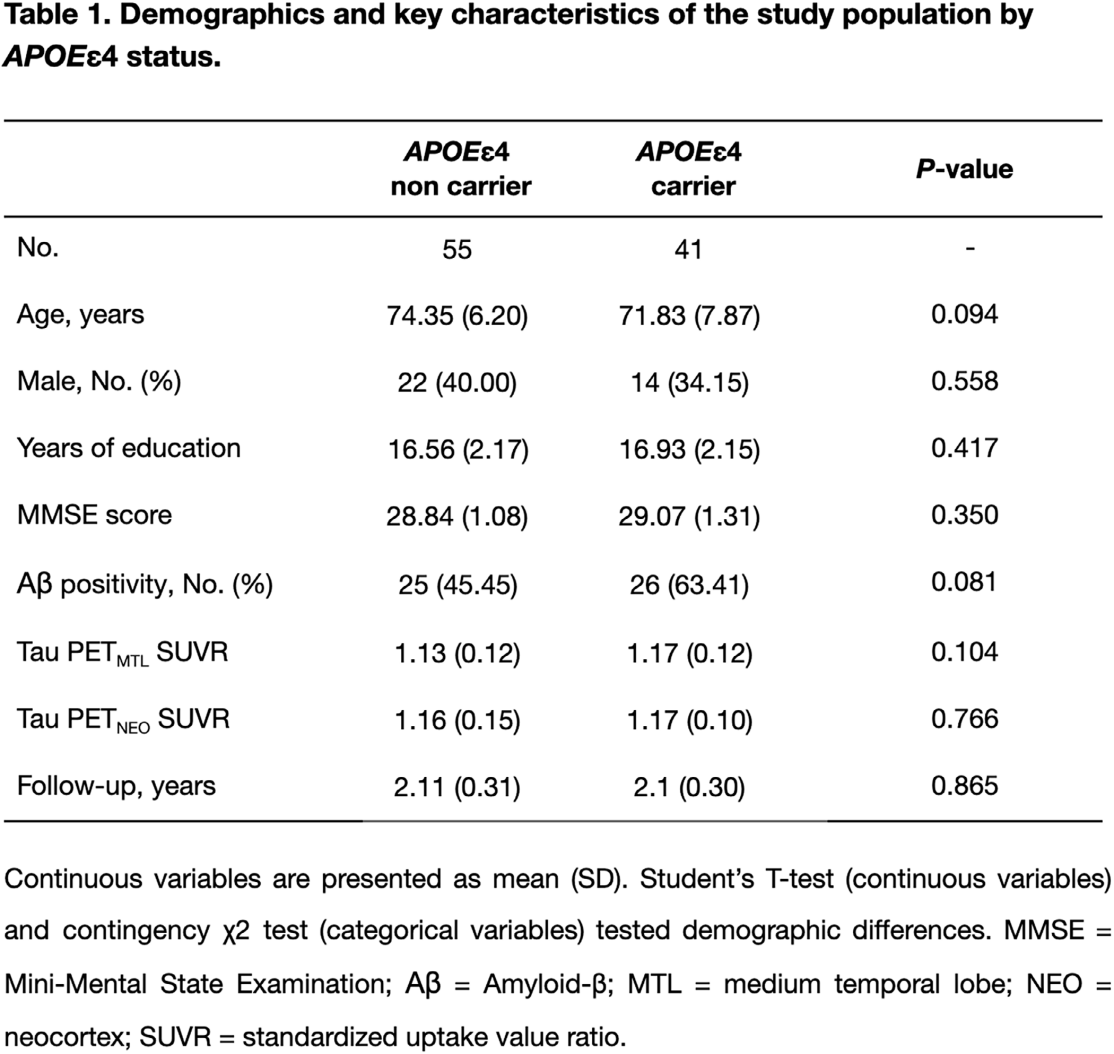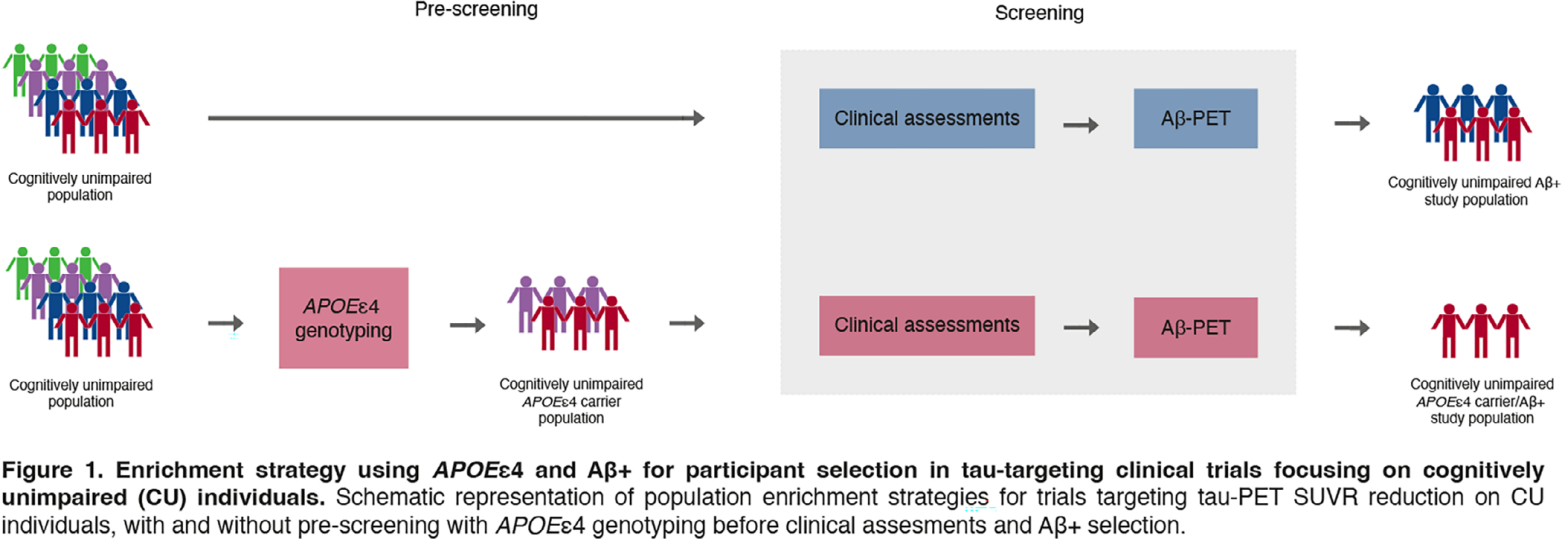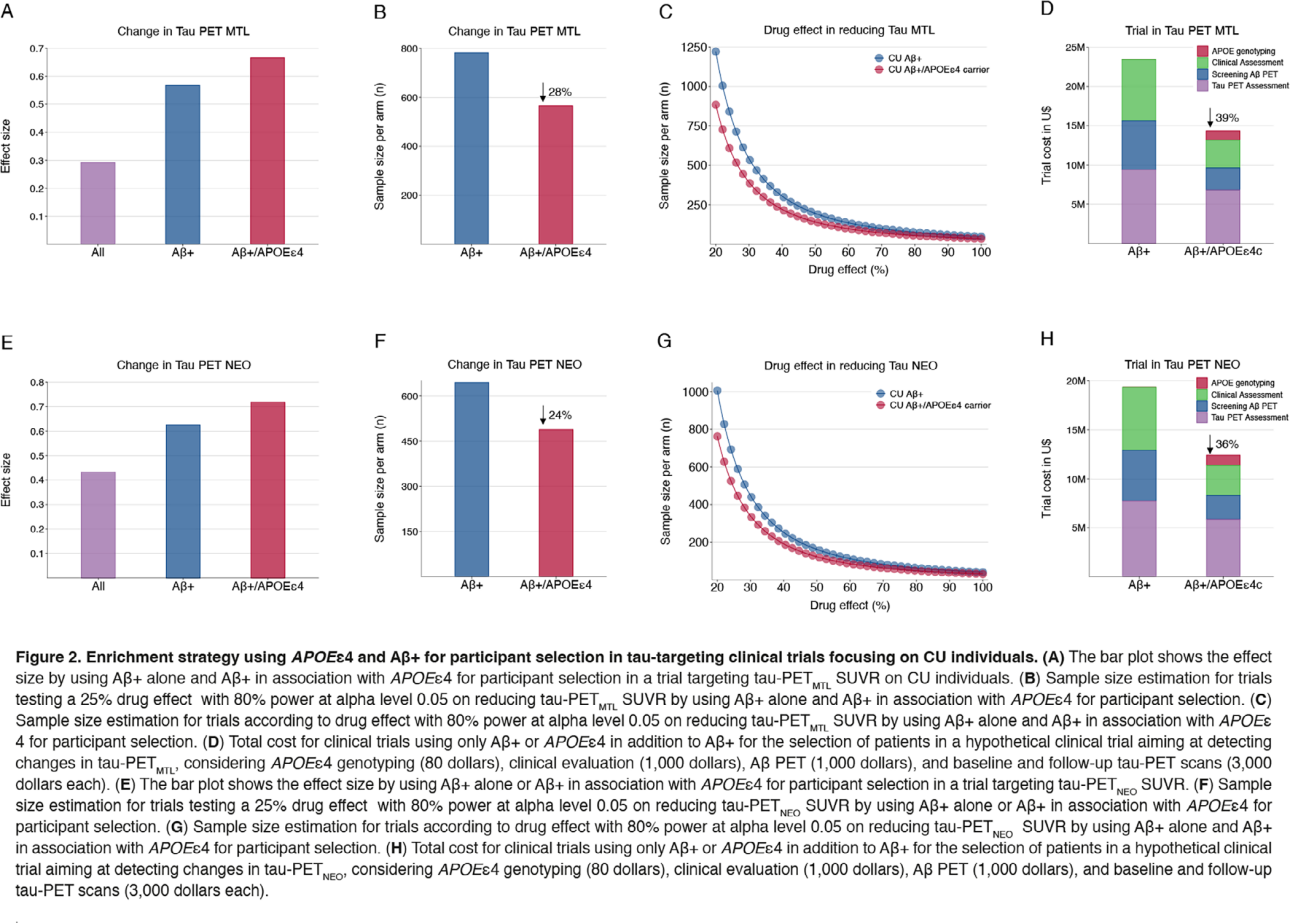# Population enrichment strategy using *APOE*ε4 genotype in tau‐targeting trials for preclinical Alzheimer's disease

**DOI:** 10.1002/alz70856_107489

**Published:** 2026-01-09

**Authors:** Laura Motter Rosso, João Pedro Ferrari‐Souza, Lucas Bastos Beltrami, Guilherme Povala, Douglas Teixeira Leffa, Firoza Z Lussier, Wagner S. Brum, Cristiano Aguzzoli, Marco Antônio De Bastiani, Andrei Bieger, Giovanna Carello‐Collar, Wyllians Vendramini Borelli, Joseph Therriault, Arthur C. Macedo, Nesrine Rahmouni, Diogo O. Souza, Bruna Bellaver, Pamela C.L. Ferreira, Pedro Rosa‐Neto, Tharick A Pascoal, Eduardo R. Zimmer

**Affiliations:** ^1^ Universidade Federal do Rio Grande do Sul, Porto Alegre, Rio Grande do Sul, Brazil; ^2^ University of Pittsburgh, Pittsburgh, PA, USA; ^3^ Universidade Federal do Rio Grande do Sul, Porto Alegre, RS, Brazil; ^4^ Neurology Department, São Lucas Hospital of PUCRS, Porto Alegre, Rio Grande do Sul, Brazil; ^5^ McGill University, Montreal, QC, Canada

## Abstract

**Background:**

Trials in preclinical Alzheimer's disease (AD) are becoming increasingly important, as AD pathological changes appear decades before dementia onset. Amyloid‐beta (Aβ) pathology and the apolipoprotein E ε4 (*APOE*ε4) carriership jointly accelerate tau tangle accumulation. However, the utility of assessing both variables to enhance participant selection for AD trials using tau positron emission tomography (PET) as outcome has not yet been explored. Here, we investigated the implications of considering *APOE*ε4 status for participant selection in tau‐targeting trials for preclinical AD.

**Method:**

We analyzed 96 cognitively unimpaired (CU) individuals (aged 57‐90 years) from the ADNI cohort that underwent clinical assessments, *APOE* genotyping, PET for Aβ ([^18^F]Florbetapir or [^18^F]Florbetaben) and tau ([^18^F]Flortaucipir) at baseline, along with a 2‐year follow‐up. Aβ positivity was determined as global [^18^F]Florbetapir SUVR >1.11 or [^18^F]Florbetaben SUVR >1.08. We calculated the sample size required for a hypothetical clinical trial testing a 25% drug effect, with 80% power at alpha level 0.05, to reduce tau‐PET accumulation in the medial temporal lobe (MTL) and neocortex (NEO), along with the total trial costs.

**Result:**

Table 1 reports the demographic information of the study population. Figure 1 shows enrichment strategies for the selection of participants in a clinical trial aiming at tau PET reduction in CU individuals. In comparison to using only Aβ positivity, the use of *APOE*ε4 genotyping together with Aβ positivity for population enrichment would reduce the sample size and total costs, respectively, by 28% and 34% in trials targeting tau PET_MTL_, and by 24% and 36%, respectively, in trials targeting tau PET_NEO_ (Figure 2).

**Conclusion:**

Our findings suggest that combining *APOE*ε4 status with Aβ positivity may be a cost‐effective strategy for enriching participant selection in AD tau‐targeting trials focusing on asymptomatic individuals, reducing required sample sizes and trial costs.